# Housing first, connection second: the impact of professional helping relationships on the trajectories of housing stability for people facing severe and multiple disadvantage

**DOI:** 10.1186/s12889-021-10281-2

**Published:** 2021-01-30

**Authors:** Rebeca D. Sandu, Frederick Anyan, Vicky Stergiopoulos

**Affiliations:** 1 Department of Psychology, University of Cambridge, Downing Street, Cambridge, CB2 3EB UK; 2 Department of Psychology, Norwegian University of Science and Technology, NO – 7491 Trondheim, Norway; 3 Center for Addiction and Mental Health, University of Toronto, Bell Gateway Building, 100 Stokes Street, Toronto, ON M6J 1H4 Canada

**Keywords:** Relationships, Housing first, Sub-groups, Housing stability, Working alliance, Severe and multiple disadvantage, Latent growth mixture model

## Abstract

**Background:**

Despite the accumulating evidence on the role of professional helping relationships for highly disadvantaged populations, methodological shortcomings have made it difficult to establish a robust relationships-outcomes link. This study sought to establish the impact of professional helping relationships on the trajectories over 24 months of housing stability for 2141 people facing severe and multiple disadvantage using data from the Housing First controlled trial in Canada.

**Method:**

The study used a mixed method design. Latent growth curve and growth mixture models assessed the impact of working alliance across the sample as a whole and within subgroups with different patterns of housing stability. Thematic analysis explored the factors that may affect the quality of working alliances within different subgroups.

**Results:**

Three distinct trajectories of housing stability emerged (i.e., Class 1: “sharp rise, sustained, and decline housing”; Class 2: “hardly any time housed”; Class 3: “high rise, sustained, and decline housing”) with professional helping relationships having different effects in each. The analysis revealed structural and individual circumstances that may explain differences among the classes.

**Conclusions:**

The findings underscore the role of professional helping relationships, as distinct from services, in major interventions for highly disadvantaged populations, and draws new attention to the temporal patterns of responses to both the quality of relationship and targeted interventions.

## Background

There is good evidence that the provision of housing can alter the trajectories of people facing severe and multiple disadvantages, particularly those experiencing homelessness. Interventions like Housing First have been shown repeatedly to reduce homelessness, increase housing stability, decrease hospitalisation, and improve quality of life [1–6]. However, the effect sizes remain modest, and there is considerable variation in the reported impact on the different populations receiving such interventions [7]. One potential influence on that variation is the quality of the relationships between the workers who facilitate the transition into housing and the people facing multiple risks. This relationship is typically part of an integrated support approach or case management (e.g., providing basic functions such as outreach, assessment, planning, linkage, monitoring, and advocacy) developed in the last three decades (see [8] for a review). Relationships vary across many domains including those that comprise Bordin’s concept of working alliance [9]: agreement about tasks and goals and the nature of the bond between client and worker.

Morse’s review [10] highlighted several service and client factors that are critical components to effective case management. Service factors include frequency of worker contact and lower lengths of time homeless. Client factors include gender of the client (women), fewer substance abuse problems and psychotic symptoms. Morse recommended that effective staff members need to have skills and abilities that enable them to develop trusting relationships with the people they support. Despite the multiple needs clients face, research has shown that people facing the greatest challenges seek first and foremost a personal connection with their workers [11, 12], facilitated by worker behaviours such as persistence, ‘going the extra mile’, being ‘like a friend’, and engaging in routine activities such as furnishing a home or going for a coffee [13–15]. Practical help is also valued in these relationships as a way to further strengthen worker-client connections [12, 16]. There is research on client and system characteristics that specifically play a role in the development of working alliances. Client characteristics that facilitate the development of positive relationships with case managers include severity of mental illness [17]; being African American, low hostility, and more perceived needs [18]; more social support, more subjective psychological problems, fewer overt psychotic symptoms, and more substance abuse problems [19]. Conversely, co-morbid substance abuse has been shown to hinder the development of client-case manager relationships [17]. System factors such as pleasant treatment surroundings, worker acts of kindness, access to independent housing have been shown to help with engagement and retention in mental and substance abuse treatment services by formerly homeless psychiatric individuals [17].

There is other evidence that the worker-client relationship can influence outcomes. Goering, Wasylenki, Lindsay, Lemire, and Rhodes [20], for instance, found that a strong working alliance was a key element in achieving housing stability for 55 homeless and severely mentally ill clients connected to a hostel outreach programme. Chinman, Rosenheck, and Lam [21] confirmed this in a sample of 2798 homeless people with severe mental illness assigned to a programme that offered outreach and intensive case management. Both research groups found that having an alliance was more beneficial than having none and that the strength of the alliance was inversely related to days of homelessness. However, Tsai, Lapidos, Rosenheck, and Harpaz-Rotem [22] found the strength and quality of the therapeutic alliance developed within the first 3 months of treatment in a supported housing programme was not associated with any major housing and employment outcomes, although it did influence subjective reports of the quality of life and social support as reported by others [23].

Several methodological limitations need to be noted, however [24]. For instance, few of the studies described above included a control group. Follow-ups were typically short. Most researchers treated their samples as homogenous whereas practitioners reported heterogeneity [25]. Recent evidence using new analytical approaches such as growth mixture modelling underlines the high variability and the complex patterns of change within this population [26]. The present paper has three aims. First, it explores the different patterns of change in housing stability over a two-year period in a population of homeless people randomly assigned to the Housing First programme in Canada. Second, it examines the impact of client-worker relationship as assessed via working alliances across the sample as a whole and within subgroups with different patterns of housing stability. Third, it explores the factors that may affect the quality of working alliances within different subgroups.

## Methods

A mixed method design was used. The study’s first two aims were assessed via quantitative analyses whereas qualitative analysis was used to explore the third aim to complement the quantitative analyses.

Three research questions were explored:

- What is the impact of the working alliance on trajectories of housing stability among homeless people, controlling for age, gender, intervention, and adverse childhood experiences? In line with other research [20–22], it was hypothesised that the quality of the working alliances would have a positive influence on housing outcomes.
- Are there multiple trajectories of housing stability among homeless people? As in previous studies [26, 27], a variety of housing stability trajectories were expected.
- How does working alliance affects subgroups of people experiencing homelessness? In light of research underscoring the role of client and systemic factors in the development of relationships, for example substance abuse or access to independent housing, it was expected that working alliance would affect different subgroups differently.

### Programme description and study procedure

The At Home/Chez Soi randomised controlled trial of the ‘Housing First’ programme took place across five Canadian cities (identifier ISRCTN42520374). The study was approved by 11 research ethics boards. Participants, recruited between October 2009 and June 2011, were stratified by the degree of mental health service support required. Those with high needs were assigned to receive either assertive community treatment (ACT) or treatment as usual (TAU) whereas those with moderate needs received intensive case management (ICM) or TAU. Goering et al. [28] have described the study protocol in detail. ACT and ICM participants received housing of their choice as well as mental health services. TAU participants had access to local services. All intervention participants were supported by a worker through an ACT or ICM team. TAU participants were directed to other local resources, but availability was subject to local resources constraints. Comprehensive in-person interviews were conducted at baseline and every 6 months, and housing was assessed every 3 months. To understand trajectories into and out of homelessness, and factors associated with each, a series of in-depth interviews were undertaken with a tenth of the sample (*n* = 195; 119 HF, 76 TAU) from the larger clinical trial. Sub-sample selection was conducted as follows. For the initial interviews, one out of every 10 participants per study condition for each site was selected by the site researchers, shifting gradually to a more purposeful selection (e.g., gender, age, ethnoracial background) to ensure representativeness of the sub-sample when compared to the larger sample at each site. Comparisons of the baseline sub-sample with the larger sample on more than 50 demographic, clinical diagnostic, and outcome measures revealed significant differences on three of these variables (more participants in the sub-sample identified as female or other, fewer had three or more symptoms on a measure of substance and more had a higher level of income in the last month than participants in the larger sample) [29]. No significant differences were detected between TAU and the HF group participants on all variables investigated. With 11% of the baseline sub-sample lost due to attrition (reasons such as not being able to locate the participant, incarceration, refusal, and death) from baseline to the 18-moth follow-up, the final sub-sample for narrative interviews included 195 participants, with the following characteristics: men (62.6%), Aboriginal people (22.9%), ethnoracial minorities (22.9%), unemployed (93.8%), not obtaining a high school diploma (56.4%), married or cohabiting (1.4%), major depressive disorder (52.6%), psychotic disorder (31.8%), alcohol dependence (34.1%), substance dependence (50.2%), age (41.3), last month’s income ($781), and lifetime months of homelessness.

Baseline consumer narrative interviews focused on life before enrolment in the programs, particularly on experiences of homelessness (e.g., how they first became homeless, life on the streets or in a shelter), mental health issues and services. Follow-up interviews focused on changes in a number of life domains since enrolment: typical day, recovery, mental health, wellbeing, relationships, material situation, housing, mental health services, including the relationships with service providers, and hopes for the future. Interviews were digitally recorded, transcribed, and stored on a Virtual Machine at St. Michael’s Hospital in Toronto. Participants were compensated for their time with C$30 to C$50. More detail about how the qualitative arm of the At Home/Chez study was developed and conducted has been published somewhere else [29].

### Participants

Participants were legal adults, absolutely or precariously housed, and had a mental disorder (with or without a concurrent substance use disorder). More than a third (34%) met the criteria for a psychotic disorder and two thirds (67%) for substance abuse or dependence. On a scale of 0–10, the average number of adverse childhood experiences was 4.6. Two thirds of the sample were males (67%), over half (57%) were aged between 35 and 54 years. Four-fifths were born in Canada and one fifth (22%) identified as Indigenous. Nearly all (93%) did not work at baseline and fewer than half (45%) had completed high school. Total number of participants included for analyses was (*N* = 2141; Females = 68%).

### Measures

The Residential Time-Line Follow-Back Inventory (RTLFB) (see [30] for reliability and validity information) was used to measure the housing stability over time by the proportion of the number of days for which any type of residence (as living in one’s own room, apartment, or house or with family) was available over the preceding 6 months. Relationships between participants and workers were assessed with the Working Alliance Inventory Short Form, adapted [31] from the original 36-item version [32] which captured the degree to which clients and workers agreed on therapy goals and tasks and the quality of the bond between them. Each of the three sub-scales has 4 items rated on a 7-point scale (1 = never, 7 = always). Because the participants’ accounts of their working alliances were assessed at multiple time points whereas the workers’ perceptions were assessed once, only the client assessments were included in the analysis. Childhood trauma was assessed using the Adverse Childhood Experiences (ACE) Scale [33] which asks 10 questions related to childhood abuse and household dysfunction before age 18 years.

### PART I: quantitative analysis strategy

Data analyses were conducted using Mplus Version 7.4 [34] and missing data were handled using full information maximum likelihood (FIML) estimation. Prior to fitting growth models, preliminary analyses examined individual trajectory plots, descriptive statistics and longitudinal covariance patterns.

#### Latent growth curve model

A well-fitting latent growth curve model (LGCM) was used to determine the overall sample trajectory. LGCM assumes a single homogenous population in which individual variations around the overall mean growth trajectory are captured by the random intercept and slope coefficients [35, 36]. Linear and quadratic growth models were used to find the best-fitting representation of change for the sample. Baseline time-invariant covariates (age, gender, intervention, adverse childhood experiences) and the time varying covariate of the working alliance measured at 6 months after baseline to the end of observation were used to determine predictors of growth factors and variation in housing stability, respectively. The following fit indices determined adequate fit: Standardized Root Mean Square Residual (SRMSR < .08), Root Mean Square Error of Approximation (RMSEA ≤ .06), Comparative Fit Index (CFI ≥ .90) and a non-Normed Fit index (NNFI; aka TLI ≥ .90) [37, 38]. The LGCM was used to select a baseline model for the growth mixture model (GMM).

#### Growth mixture model

The Latent growth mixture model (LGMM) [39] framework was used to assess heterogeneity in patterns of change. The LGMM approach adopted allows for differences in growth parameters across unobserved subgroups or classes [36] whereas LGCM assumes all individuals belong to a single population. Additionally, LGCM assumes that covariates affecting class membership influence everyone in the same way. We hypothesised that qualitatively different subgroup trajectories may exist in the sample of homeless people reflecting variations in their health, impairment, and resilience. Progressively larger numbers of latent class (1- class to 4-class) solutions were run to determine the optimal number of classes [39–41]. To determine the optimal class solution, a variety of fit statistics with classification accuracy were examined, so that average probability of belonging to the most likely class should be high, and the average probability of belonging to the other class should be low [42]. In particular, the Bayesian Information Criterion (BIC), sample-size adjusted BIC (ABIC), Lo-Mendell-Rubin Likelihood Ratio test (LMR-LRT), Bootstrapped Likelihood Ratio test (BLRT), Akaike information criterion (AIC) indices and the Entropy values were examined. The fit indices in combination with theoretical interpretability and class profile plots guided the final model selection. Once the optimal class solution was selected, the R3STEP [43] approach was used to include covariates (age, gender, intervention, adverse childhood experiences) in the model to predict class membership. R3STEP results in less biased parameter estimates while maintaining a stable class solution and interpretable coefficients for the covariates [43]. Working alliance was included in the model as a time varying covariate to explain variation in housing stability.

### PART II: qualitative analysis strategy

In this study, a tenth of the 195 available qualitative interviews (*n* = 20) at follow-up was randomly selected to enable a deeper understanding of results within two of the three classes with different trajectories of housing stability selected from the quantitative analyses. Ten participants from each class, varied with respect to age, intervention mode, gender, and number of adverse childhood experiences were randomly selected (see Table 1). Availability of transcripts in English explains why one of the five sites (Toronto) is overrepresented in this sub-sample.

**Table 1:** Characteristics of the qualitative sub-sample

Participant	Age range	Gender	Levels of need	Intervention	Class	Site
1	18–24	F	moderate need	ICM	1	Toronto
2	25–34	F	moderate need	TAU	1	Vancouver
3	18–24	F	high need	ACT	1	Moncton
4	45–54	M	high need	ACT	1	Moncton
5	25–34	F	high need	ACT	1	Moncton
6	25–34	F	high need	TAU	1	Moncton
7	45–54	M	high need	ACT	1	Toronto
8	25–34	M	moderate need	ICM	1	Toronto
9	45–54	M	high need	TAU	1	Toronto
10	35–44	M	high need	ACT	1	Toronto
11	35–44	M	high need	ACT	2	Toronto
12	45–54	M	moderate need	ICM	2	Toronto
13	45–54	M	high need	TAU	2	Toronto
14	25–34	M	moderate need	TAU	2	Toronto
15	18–24	F	high need	TAU	2	Winnipeg
16	45–54	F	moderate need	TAU	2	Vancouver
17	25–34	M	moderate need	TAU	2	Vancouver
18	35–34	F	high need	ACT	2	Vancouver
19	25–34	F	moderate need	ICM	2	Toronto
20	45–54	F	moderate need	ICM	2	Vancouver

Three questions guided the qualitative analyses: (i) do participants in the two classes differ in their perceptions of relationship quality, (ii) do structural factors in programme implementation play a role in the development and quality of relationships between workers and participants, and (iii) what factors could explain group differences in the way working alliance impacted housing trajectories?

The analysis was informed by a critical realist approach [44], which assumes there is ‘truth’ in the data that needs uncovering. The first question adopted a naïve realist approach, taking at face value how participants described healthy relationships; for the remaining questions, researchers were guided by a critical realist approach, taking the position of detectives and using their skills, knowledge, and experience to interpret the views of participants and understand differences between classes.

The analysis employed Braun and Clark’s [45] six-stage thematic analysis approach (familiarise with data; generate code; search for themes; review themes; define and name themes; and write report). The analysis focused on the sentences in which the relationships with workers were mentioned. Although the research questions that resulted from the quantitative analysis guided the coding, no list of pre-defined codes was used. Codes were generated at each step. Semantic and latent themes were coded. The analysis was conducted using the qualitative software NVivo 12. The trustworthiness of the emerging findings was tested twice. First, the author kept a reflexive journal in which noteworthy aspects of the analysis were recorded. Second, an external auditor (MSc student) scrutinised a third of the data and the emerging findings, generating further insights. For example, additional dimensions (e.g., care, honesty) valued by participants in good relationships were revealed by the audit.

## Results

### Quantitative analyses

#### Latent growth modelling (unconditional model)

A linear growth trajectory of housing stability showed poor fit (*χ*^*2*^ *=* 1044.753, *df* = 9 *p* < .001; SRMR = .269; RMSEA = .232, [90% CI = 0.220, 0.244]; CFI = .629; TLI = .588). A quadratic growth trajectory was better, reaching acceptable fit (*χ*^*2*^ *=* 240.709, *df* = 5, *p* < .001; SRMR = .064; RMSEA = .148, [90% CI = 0.133, 0.165]; CFI = .916; TLI = .831). The RMSEA tends not to perform well with growth curve models because of the few degrees of freedom [46]. The average intercept or initial status was significant (I = 8.404, *p* < .001), and the linear trajectory, which showed a positive rate of change, was also significant (S = 36.340, *p* < .001). The quadratic growth factor declined significantly over time, showing a decelerating change in growth (Q = − 6.899, *p* < .001). The variance of the intercept was not significant (67.979, *p* = .085), which indicated that there were no inter-individual differences in the initial status of housing stability. The variance in the linear (823.691, *p* < .001) and quadratic (40.476, *p* < .001) growth factors were highly significant, indicating significant inter-individual differences in the rate of change.

#### Latent growth modelling with time-invariant and time varying covariates

Including the time-invariant covariates (LGCM – TIC) did not improve model fit (*χ*^*2*^ = 312.424, *df* = 12, *p* < .001; SRMR = .042; RMSEA = .128 [90% CI = 0.116, 0.140]; CFI = .890; TLI = .725). Another conditional model with the time-invariant covariates and working alliance as a time varying covariate (LGCM –TIC and TVC) (*χ*^*2*^ = 66.786, *df* = 28, *p* < .001; SRMR = .032; RMSEA = .049 [90% CI = 0.034, 0.065]; CFI = .938; TLI = .889) showed adequate fit. Parameter estimates for both LGCM – TIC and LGCM – TIC and TVC models are shown in Table 2.

**Table 2:** Parameter estimates for LGCM with time-invariant (TIC) and time varying covariates (TVC)

	LGCM-TIC			LGCM-TIC and TVC		
	*Est*	*S. E*	*p*	*Est*	*S. E*	*p*
Intercept mean	10.613	1.418	.000	3.890	3.055	.203
Slope mean	17.238	2.423	.000	19.060	6.245	.002
Quadratic mean	−2.454	2.423	.000	−2.224	1.739	.201
Intercept variance	35.157	47.684	.461	23.847	57.460	.678
Slope variance	247.302	73.600	.001	401.945	94.972	.000
Quadratic variance	12.626	4.608	.006	25.393	6.184	.000
Intercept with Slope	29.548	42.883	.491	−11.291	52.458	.830
Intercept with Quadratic	−9.657	8.390	.250	0.389	10.674	.971
Slope with Quadratic	−48.948	17.710	.006	− 96.970	23.499	.000
Intercept predicted by						
Age	−0.056	0.047	.225	0.022	0.076	.767
Gender	−3.307	1.104	.003	−1.405	1.706	.410
ACE	−0.342	0.173	.048	−0.304	0.275	.268
Intervention	2.528	1.092	.021	5.912	2.696	.028
Slope predicted by						
Age	0.204	0.079	.010	0.126	0.124	.308
Gender	−1.212	1.886	.520	2.134	2.788	.444
ACE	0.074	0.297	.802	0.142	0.450	.752
Intervention	38.412	1.857	.000	37.599	4.400	.000
Quadratic predicted by						
Age	−0.027	0.020	.173	− 0.009	0.032	.777
Gender	0.059	0.471	.901	−0.326	0.726	.654
ACE	−0.032	0.074	.665	−0.030	0.117	.799
Intervention	−8.670	0.463	.000	−9.101	1.141	.000
Housing stability predicted by						
T2: Working alliance				0.281	0.044	.000
T3: Working alliance				0.026	0.053	.623
T4: Working alliance				−0.010	0.052	.842
T5: Working alliance				0.025	0.092	.789

*Note: LGCM-TIC* Latent growth model with time-invariant covariates; *LGCM-TIC* and *TVC* Latent growth model with time-invariant and time varying covariates; *ACE* Adverse childhood experiences

**Fig. 1:**
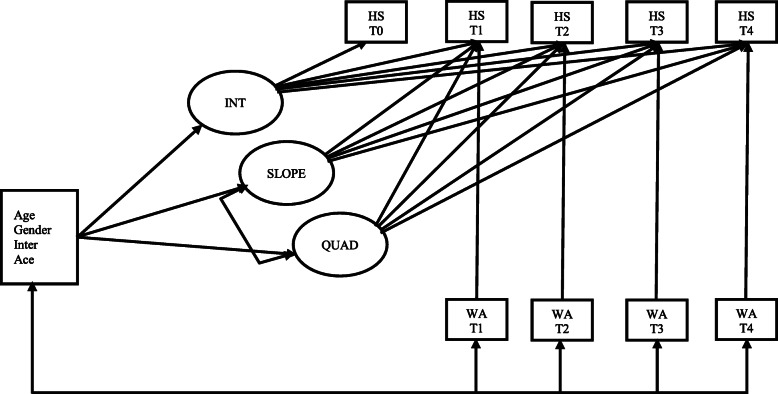
LGCM with time invariant and time varying covariates. *Note:* Inter = Intervention; Ace = Childhood adverse experiences; INT = Intercept; SLOPE = Linear growth factor; QUAD = Quadratic growth factor; HS = Housing stability; WA = Working alliance. Nonsignificant covariance between Intercept and Slope, and Intercept and Quadratic growth factors not shown

Figure 1 shows the LGCM – TIC and TVC. The variance in the linear (401.945, *p* < .001) and quadratic (25.393, *p* < .001) growth factors, and their covariance (− 96.970, *p* < .001), were highly significant, indicating significant inter-individual differences in the rate of change as a function of the covariates. The *R*^*2*^ values (i.e., explained variance) in housing stability (baseline = 7.5%; six months = 35.2%; twelve months = 66.4%; eighteen months = 50.1%; twenty-four months = 34.2%) indicate that variation in housing stability is well explained by the growth factors and working alliance. Controlling for the effect of working alliance, the time-invariant covariates explained (*R*^*2*^ = 24.7%, *p* < .001) in the linear and (*R*^*2*^ = 22.8%, *p* < .001) in the quadratic growth factors, but not the intercept (*R*^*2*^ = 14.2%, *p* = .660). Participants who received the intervention experienced a rise but also a decelerating growth in housing stability. The working alliance had a significant positive effect on housing stability at 6 months, but the impact gradually declined from the twelfth month, although the declines were not statistically significant.

#### Growth mixture model of housing stability

Because the unconditional quadratic growth trajectory demonstrated acceptable fit to the data, it was decided to estimate the growth mixture model to determine whether subgroups of individuals could be identified within the data. The latent class growth analysis (LCGA), which freely estimates class means but fixes within-class variances to zero, assuming within-class homogeneity was used. Because estimating the LGMM produced negative variances, growth factors’ variances were fixed to zero for the LCGA [47]. Further, because invariance of intercept means was not significant (*χ*^*2*^ = 2.487, *df* = 2, *p* = .288), the intercept was constrained to be equal for all classes. Non-invariance tests in linear (*χ*^*2*^ = 4738.001, *df* = 2, *p* < .001) and quadratic (*χ*^*2*^ = 2249.947, *df* = 2, *p* < .001) growth factors showed that the linear rate of change and the decelerating change were significantly different between classes.

Table 3 shows model fit indices for all unconditional mixture models under comparison. The model with the 1-class solution showed the largest AIC, BIC and ABIC values, indicating its fit was worst. In addition, the LMR LR test, ALMR LR test and BRLRT in the 2-class solution all had *p*-values < .05, suggesting that it was appropriate to reject a single-class solution in favour of at least two classes. The results suggested that the rate of change in housing stability among the participants was heterogenous, not homogenous, even though participants had similar initial status. To determine the optimal number of classes, we examined the Bayesian Information Criterion (BIC) and the Lo-Mendell-Rubin Likelihood Ratio test (LMR-LRT), the Bootstrapped Likelihood Ratio test (BLRT) and guidance by theoretical interpretability of the class solution. Statistically significant *p*-values for the LMR-LRT and BLRT indicated that the current (k-class) model fit better than the model with one less class (*k*-1 class). The LMR-LRT of the 4-class solution indicated that it did not fit better than the 3-class solution, and the 3-class solution’s BIC was smaller than that for the 2-class solution. Alternatively, when comparing classes, there was clear improvement in model fit when moving from a 1-class to a 2-class solution, and the LMR-LRT suggested improved model fit when moving from a 2-class to 3-class solution, but reduced model fit when moving from a 3-class to 4-class solution. Thus, the 3-class solution that showed a reasonable representation of the data and a more parsimonious model was selected.

**Table 3:** Model Fit Indices for Mixture Model Analysis of Housing Stability

	AIC	BIC	ABIC	Entropy	LMR LR Test *p*-values	ALMR LR Test *p*-value	Sample proportion per class	Classification accuracy	BLRT *p*-value
1-Class	97,888.464	97,939.485	97,910.891	–			2141	–	–
2-Class	93,144.013	93,212.041	93,173.916	.944	*p* < .001	*p* < .001	(1304; 61%), (837; 39%)	.979–.984	*p* < .001
3-Class	91,663.649	91,748.684	91,701.027	.948	*p* < .01	*p* < .01	(1240; 58%), (794; 37%), (107; 5%)	.974–.979	*p* < .001
4-Class	90,635.439	90,737.482	90,680.293	.951	.466	.475	(1203; 56%), (93; 5%), (778; 36%), (67; 3%)	.972–.987	*p* < .001

*Note. AIC* Akaike information criterion; *BIC* Bayesian information criterion; *ABIC* Sample-size adjusted BIC; *LMR LR* Vuong-Lo-Mendell-Rubin Likelihood Ratio Test; *ALMR LR* Lo-Mendell-Rubin Adjusted LRT Test; *BLRT* Bootstrap likelihood ratio test

**Fig. 2:**
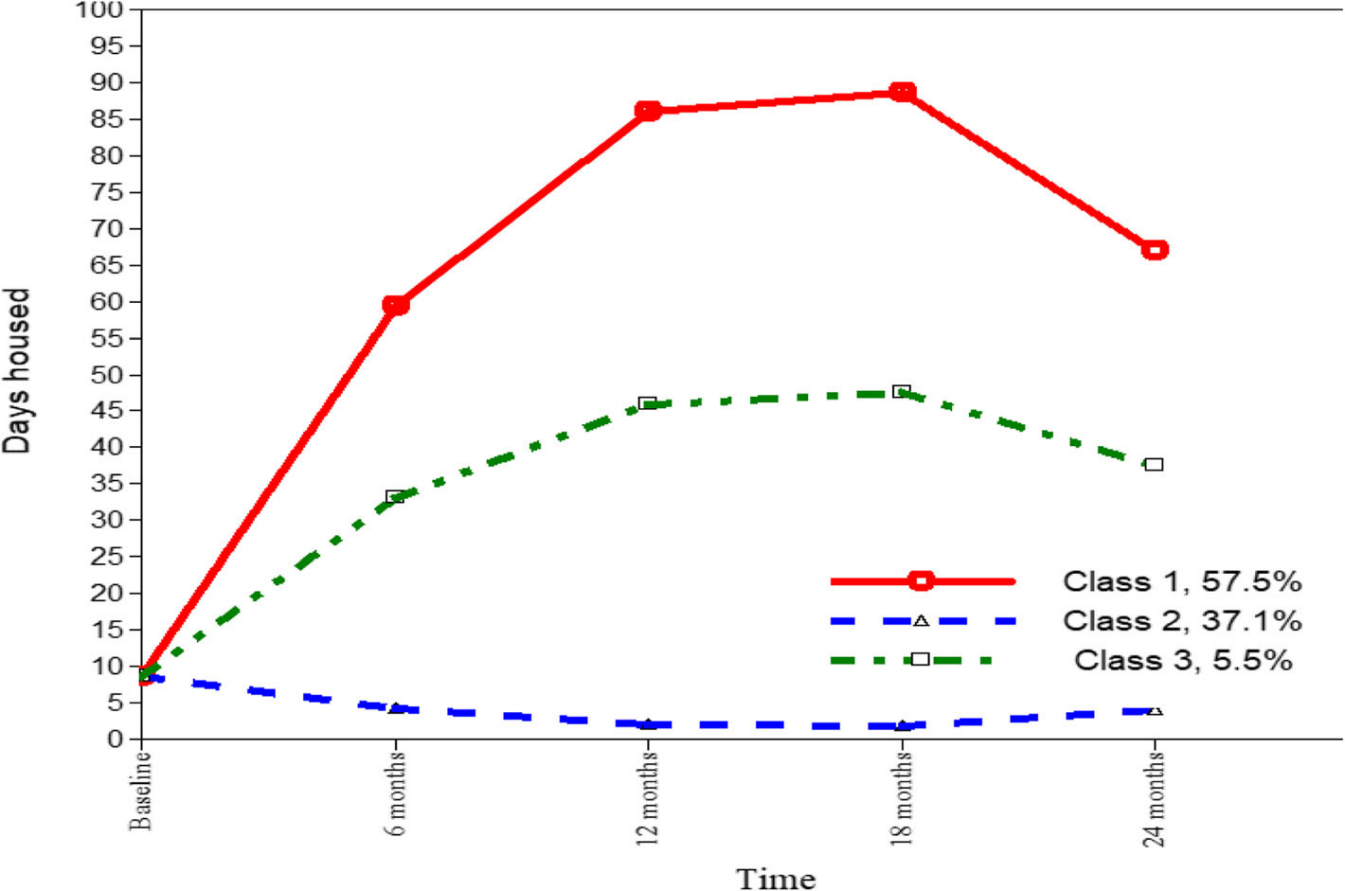
Three-class profile plot of housing stability. *Note:* Class 1: Sharp rise, sustained, and decline housing; Class 2: Hardly any time housed; Class 3: High rise, sustained, and decline housing

Figure 2 displays the class profile plot and Table 4 displays the growth parameters. Class 1 showed “sharp rise, sustained and decline housing,” with sharp initial increases and decelerating rates of change. Class 2 showed “hardly any time housed” and Class 3 “high rise, sustained and decline housing” in housing stability.

**Table 4:** Growth parameters for each class

Class #	Intercept (SE)	Linear slope (SE)	Quadratic slope (SE)
1	8.645*** (0.548)	62.739***(0.730)	− 12.039*** (0.218)
2	8.645*** (0.548)	−5.554*** (0.779)	1.100*** (0.233)
3	8.645*** (0.548)	30.050*** (3.694)	−5.710*** (1.056)
*** *P* < .001			

#### Predictors of class membership

Once the growth mixture model was established, the R3STEP approach in Mplus was used to examine predictors of class membership. Housing stability was regressed on working alliance from T1 to T4 in each class. The reference category was switched across regressions so that all pairwise comparisons were made. Age and intervention at baseline were significantly related to class membership with respect to the high rise, sustained and decline housing and sharp rise, sustained and decline housing classes (see Table 5).

**Table 5:** Logistic regression parameters predicting class membership

Reference Class = High rise, sustained and decline housing	High rise, sustained and decline housing	Hardy anytime housed
Hardly anytime housed		
Age	0.009 (0.020)	–
Gender	0.303 (0.571)	–
Intervention	1.472 (0.766)	–
Childhood adverse experiences	−0.102 (0.094)	–
Sharp rise, sustained, and decline housing		
Age	0.033** (0.011)	0.024 (0.018)
Gender	−0.002 (0.260)	−0.305 (0.553)
Intervention	2.072*** (0.314)	0.600 (0.767)
Childhood adverse experiences	−0.066 (0.043)	0.036 (0.087)

**p < .01; ***p < .001

In order to compare the means for age and intervention status across the classes, the DU3STEP approach, which assumes unequal means and variances, was used in a separate analysis. The results showed that older participants and those receiving the intervention were more likely to be in sharp rise, sustained and decline housing, than younger persons or those not receiving intervention would be. All other pairwise comparisons were nonsignificant. The working alliance differentially predicted housing stability across the three classes. In the *sharp rise, sustained and decline housing* class, the working alliance significantly predicted housing stability at six (*b* = 0.228, *p* < .001) and twelve (*b* = − 0.081, *p* < .05) months, but not at eighteen (*b* = 0.001, *p* = .925) and twenty-four (*b* = − 0.031, *p* = .674) months. In the *hardly anytime housed* class, the working alliance did not predict housing stability over time, indicating that working alliance did not have an effect on housing stability for participants in this class. In the *high rise, sustained and decline housing* class, the working alliance predicted housing stability at six (*b* = 0.304, *p* < .05), twelve (*b* = 0.278, *p* < .05), and twenty-four (*b* = 1.007, *p* < .001), but not eighteen (*b* = − 0.011, *p* = .734) months. The class varying results imply that class membership moderated the causal relationship between working alliance and housing stability when controlling for other covariates.

### Qualitative analyses

The quantitative analysis identified three patterns of associations between working alliance and housing stability. In groups one *(“sharp rise, sustained and decline housing”)* and three *(“high rise, sustained and decline housing”)* there was a strong relationship between working alliance and housing stability, and in group two *(“hardly any time housed”)* working alliance did not predict housing stability. To complement the quantitative analyses, we explored three sub-questions with a qualitative approach, by analysing interview transcripts of 10 participant from the *sharp rise, sustained and decline housing class* (i.e., working alliance predicted housing stability) and 10 from the *hardly anytime housed* class (i.e., working alliance did not predict housing stability). The findings indicated that participants in both classes held similar perceptions of what counted as a good relationship with their worker. They wanted those assigned to help them to be available when needed, able to listen, authentic, and caring as well as to provide practical help. They sought personal connections that went beyond professional transactions.

If I really need her yeah, she’s there [Yeah] you know, I can call her and there’s times where I have really needed her and she like drove to my place and [Awesome] or gave me rides to appointments, she’s an amazing worker. (Female, 18-24, Intervention, 9 ACEs, from *sharp rise, sustained and decline housing class*).

Very much so, it’s probably one of the, one of the most, most reasons why I am still where I am at because of them and the support [Um hmm] you know what I mean, [Yeah] if that makes any sense. […] Um hmm, and real support, not just phony going through the motions shit. (Male, 45–54, Intervention, 5 ACEs, from *hardly anytime housed class*).

Workers considered by participants to be unhelpful were described as infrequently available, not trustworthy, impersonal, judgmental, and avoidant of the personal choices made by those they helped.

She [worker] was in my space, she just kept coming to my door and I’m sick of them you know, they pay attention to ya if you flood or burn the house down. But not otherwise, not. (Female, 18–24, Intervention, from *sharp rise, sustained and decline housing class*).

My old [worker] um closed my file while I, while I was still on uh probation, and told me that I didn’t have enough goals to work on yet. (Female, 18–24, Intervention, 0 ACEs, from *hardly anytime housed* class).

Additional findings revealed that four structural factors appeared to explain the variation in relationship quality across the two groups: staff turnover, timing of worker connection to participant, relational capability of the workers, and the uncertainty associated with the end of the programme (which may also explain variations in the growth curve analysis).

Frequent staff changes meant that some participants had to continually re-tell their personal stories. In some cases, this led to disengagement.

I just need somebody and she tells me she’s not qualified and that’s it. I usually just don’t answer the door, I peek out and see who it is, and I don’t answer the door. Because why would I want to repeat the same story to a different person. (Female, 25–34, Intervention, 1 ACE, from *sharp rise, sustained and decline housing class*).

Comparing the quantitative records of worker assignments to participants with the latter’s accounts of their relationships with those workers suggests that delay made it harder to form positive relationships.

Participants indicated that workers varied across sub-groups in their relational capability, including their ability to listen, provide practical help, and invest the time needed to form a connection that went beyond the minimum required by the service.

The end of programme was a continual threat to intervention participants in particular. It was a reminder that the relationship with the worker was to some extent a function of the intervention and could not endure, evoking feelings of sadness. This was compounded by uncertainty about their housing situation and support services.

My new family now. I’m gonna miss it once it’s finished you know. (Male, 35–44, Intervention, 6 ACEs, from *sharp rise, sustained and decline housing class*).

I have accumulated a lot of stuff from my apartment, a lot of nice stuff and I love my apartment and it’s going to be {Long pause} hard leaving it if they don’t get more funding after the 3 years. (Male, 35–44, Intervention, 5 ACEs, from *sharp rise, sustained and decline housing class*).

The above themes applied across classes. Differences between the groups, and in particular, on why the working alliance had little effect on participants’ housing stability in the *hardly anytime housed* class was further explored. In this class, a higher proportion of participants received TAU (66%) compared to the *sharp rise, sustained and decline housing* class (41%), decreasing the chances of them being connected to a worker (See Table 5).

Like, I’d feel like I was getting support in one area and [I would wonder] God, how long is it going to last? You know, I had different people that I’d see on a regular basis, but people were always passing the buck. And it got to the point for me where it didn’t matter who I was talking to – I didn’t feel that I was getting the right answers. I would feel like I was spinning my wheels. (Female, 45–54, TAU, 7 ACEs, from *hardly anytime housed* class).

In addition, the qualitative analyses showed that participants from the *hardly anytime housed* class actively avoided social relationships. Contact with family and friends was also less. In the *sharp rise, sustained and decline housing* class, by contrast, relationships with family and friends were often cited as sources of support.

No contact with family? They’re all dead. My sister I haven’t seen in 25 years and that’s about it. (Male, 45–54, TAU, 4 ACEs, from *hardly anytime housed* class).

A lot of my friends are disabled and just can’t hold the alcohol and my family members we’re distant. I talk to them once a year. (Male, 25–34, TAU, 5 ACEs, from *hardly anytime housed* class).

Other participants in the *hardly anytime housed* class reported past high consumption of drugs and involvement with the criminal justice system and these risks endured throughout the study period.

When I left (Place08), I had to go before I lost it and put myself into jail for a longer period of time, so I had to leave the building. I was only in there from February to June. That’s as much as I could take of it. (Male, 23–34, TAU, 7 ACEs, from *hardly anytime housed* class).

Well when I went into the (Place03) I had 6 months clean. I managed to stay 2 months clean doing the laundry there... And then the building started filling up, and it was filling up with all the worst druggies – the people that really were un-housable anywhere else. And they started knocking on my door asking for lighters at three o’clock in the morning, offering me tokes... I mean, it was just beyond... It’s like, they could see I was straight and they liked that, but they wanted to bring me down. So, I had a lot of people offering me drugs and da-da-da-da-da. And I finally fell. (Female, 45–54, Intervention, 2 ACEs, from *hardly anytime housed* class).

In addition to the risks to housing stability, interviews with the *hardly anytime housed* class participants made more references to guilt resulting from their perceptions of the way in which their behaviours negatively affected others, their families included. As one participant put it, a calm space afforded by a house, provided with the intention of creating stability, allowed feelings of guilt to surface, so undermining housing stability.

I don’t know if it’s all (I/A), all the shits that’s been coming down the last 3 months or if it’s my past catching up with me. {Long pause} But I lived a really, really fucked up life eh [Um hmm], I did (I/A) like I was a mess worse than so now, worse than now [Um hmm] pardon me [Um hmm], more so than now. {Long pause} For 7 years in (I/A) penitentiary watching people dying by the knife blade and [Yeah] hockey sticks and having nightmares and shit at night eh? {Long pause} I don’t need this in my life right now when this is (I/A), things are starting to get back on track, it starts happening to me. (Male, 45–54, Intervention, 5ACEs, from *hardly anytime housed* class).

## Discussion

This study investigated the impact of professional helping relationships on the trajectories over 24 months of housing stability for people facing severe and multiple disadvantage using a mixed-method design. Overall, the quality of the client-worker relationship made a significant contribution to the participants’ housing stability. The population was not homogenous. Three distinct trajectories of housing stability emerged (i.e., Class 1: “sharp rise, sustained and decline housing”; Class 2: “hardly any time housed”; Class 3: “high rise, sustained and decline housing”) with professional helping relationships having different effects in each. The analysis revealed structural and individual circumstances that may explain differences among the classes. Not everyone had an equal chance of remaining stably housed: specific subgroups of people facing severe and multiple disadvantage face unique and different adversities so that interventions are not equally effective for different subgroups of homeless people. At the start, being male, the number of adverse childhood experiences, and being in the treatment-as-usual group reduced tenancy security. Older participants who received the intervention were more likely to remain in their homes over the study period. However, when the strength of the working alliance between the worker and participant was also considered, the above variables no longer predicted housing stability, demonstrating the importance of relationship quality.

There is overlooked heterogeneity in the recovery profiles of highly disadvantaged individuals (see [26] for exceptions). The first of three classes identified in this study had 1240 members (57.5% of the sample, with 73.6% of them receiving the intervention) (See Table 6). Participants in this group (who were older, disproportionately female, and with adolescent-onset homelessness) started with an initial spike in housing stability that was maintained until the last 6 months of the study when it diminished. The second group, representing over a third of the sample (37.1%, with 33.4% of them receiving the intervention), were the least successful in achieving housing stability and spent almost all the time unhoused. Participants in this group were predominantly male (73%), had longer histories of being homeless, could count on fewer people for social support, and spent a higher proportion of the study period imprisoned than did participants in the other groups. The third group and smallest group (5.5%, with 53.3% of them receiving the intervention) followed a similar pattern of housing stability as the first group. Participants were older when they first became homeless and spent fewer years being homeless, relied on larger social networks, and had higher rates of substance use than participants in the first group (comparable to that of participants in second group).

**Table 6:** Class characteristics identified with latent class growth analysis

Variable	Sharp rise, sustained and decline housing	Hardly anytime housed	High rise, sustained and decline housing
TAU, n (%)	327 (26.4)	529 (66.6)	50 (46.7)
HF, n (%) HF ICM, n (%)	913 (73.6) 587 (64.3)	265 (33.4) 161 (60.8)	57 (53.3) 30 (52.6)
Age (years)	41	39	39
Gender (%)			
Female	35	27	29.9
Male	65	73	70.1
Education (%)			
< High school	53.1	61.6	56.1
Completed high school/some higher education	19.4	16.9	21.5
Completed trade school/ undergraduate	27.4	21.5	22.4
Age first time homeless	16	27	31
Total time homeless (years)	5.9	6.8	3.9
Childhood trauma (Ace total score)	4.3	4.7	4.2
Diagnosis (MINI %)			
Depression	51.3	51.4	52.3
Mood disorder with psychotic features	16.1	16.9	14
Psychotic disorders	35.4	38.8	35.5
Panic disorder	22.3	24.1	24.3
Manic or hypomanic Episode	12.8	13.6	15
PTSD	29.6	28.5	26.2
Baseline mental illness			
severity (CSI cut-off, %)	75.9	75.1	74.5
Social network (MCAS %)			
Nobody (baseline)	6	5.4	3.7
Nobody (6 months)	2.2	5.1	3.3
Nobody (12 months)	1.6	5.8	1.1
Nobody (18 months)	1.2	5.9	1.1
Nobody (24 months)	0.7	2.4	0.2
Time in prison during study (%)			
6 months	1.5	7	3.7
12 months	1.12	9.6	2.8
18 months	0.31	12.46	3.89
24 months	1.13	10.24	8.03
Substance problems (GAIN SPS high use, past year)			
12 months	36.9	46.2	46.7
24 months	28.5	35.5	43

*Note: ACE* Adverse Childhood Experiences; MINI = Mini-International Neuropsychiatric Interview; *CSI* Colorado Symptom Index; *MCAS* Multnomah Community Ability Scale (social network item); *Gain-SPS* Global Assessment of Individual Need Substance Problem Scale (only high use reported here); *TAU* Treatment as Usual; *ICM* Intensive Case Management

The non-linear patterns of housing stability may be important, as suggested by Adair et al. [26] too. Policy makers and intervention scientists think programmatically in one- or two-year blocks, but the disadvantage that qualifies participants for intervention is accumulated over a lifetime. Moreover, as many participants in the intervention group expressed anxiety during the second half of the study, in the context of uncertainty about program sustainability, it is important to attend to smooth programme endings and transitions to promote housing stability among recently housed individuals. Indeed, at the study end, 75% of the programmes were sustained, and the majority of participants maintained their housing [48].

There was significant variation both in the impact of the intervention and in the contribution of the working alliance between support workers and participants. Relationships in the first year predicted housing stability over time for one group (Class 3), in the first year for another group (Class 1), and not at all for a third group (Class 2). Research has shown that an early onset of homelessness is associated with numerous childhood problems [49], psychiatric comorbidities and substances use [50]. The fact that working alliance impacted positively participants from Class 1–– who became homeless in their adolescence rather than adulthood as participants from Class 2 and 3–– indicate the potential for good of relationships for the most vulnerable populations.

The qualitative analyses revealed a number of possible reasons for this class variation. In line with previous work [17, 19], individual circumstances played a role in relationship development and housing instability. Participants’ lifestyles (drug use) may make it harder for them to form relationships and easier for those relationships to be disrupted. Spending more time in prison, as it was the case with participants in Class 2 compared to those in Class 1, might have affected relationships by reducing the amount of time available for workers and participants to meet while also adding to housing instability.

Counter-intuitive impacts were found. A stable home can produce time for reflection, bringing to the present guilty feelings about the impact of past behaviours on family and friends. As Sandu [2019, unpublished data] found, people facing severe and multiple disadvantage have an emotional reaction to their circumstances, which coupled with a distrust of others resulting from a history of poor relationships leads them to back away from potential relationships and supports. Depletion and active avoidance of social connection can make it harder to form and sustain the effects of positive professional relationships. Padgett, Henwood, Abrams, and Drake [51] also found that people who experienced serious mental illness, substance abuse, and homelessness used a “loner talk” when talking about themselves in relation to others. However, Padgett et al. [51] also revealed that the participants had a desire to connect with others but were impeded by other factors, such as ambivalent nature of family ties, the focus on achieving housing stability rather than relationships, negative social networks, and substance abuse. These findings underscored the role of the relationships, as distinct from services, in major interventions for highly disadvantaged populations, and draws new attention to the temporal patterns of responses to both the quality of relationship and targeted interventions.

This study also explained significant variations among sub-groups of participants and elucidated the characteristics of high-quality relationships between workers and participants, underlining the importance of a sense of personal connection [11, 12, 14, 15]. Relationships that prioritise building a personal bond before risk resolution have been shown to change trajectories by changing how people feel about themselves (e.g., feeling valued, worthy), cognition (disrupting negative patterns of thinking), and agency (recovering a sense of ‘I can do’) [52]. Other research has shown the positive associations between working alliance and community integration (physical and psychological) and quality of life for this population [53]. Study participants understood what counted as a quality relationship, but there was considerable variation in their (and their workers’) ability to secure such relationships. Temporal aspects of these relationships were also highlighted. The intervention — the provision of housing — endured but the change of workers was sometimes experienced by people as a sense of personal loss [51], and which contributed to housing instability.

There are several limitations to the study. The concept of the working alliance, although widely used, fails to fully capture the essence of worker-client relationships. Sub-components of the working alliance were not identified, although this could have resulted in further understanding of how working alliances function in this population. Inclusion of different outcomes, in addition to housing stability, could have yielded a fuller picture of how relationships affect outcomes. Estimation of the mixture model did not use predefined group membership that could identify different trajectories for intervention and treatment-as-usual groups, although in the only study that has done this [26], the best performing class was similar to the one found in this study. The R3STEP analyses avoided biased class formation when including covariates by retaining original class membership but the predictors’ coefficients may have been biased by the R3STEP procedure, which does not allow missing cases on exogenous predictors. Causal relationships between covariates and class formations should thus be inferred with caution. The themes provided by the qualitative analysis could have been further explored by increasing the number of transcripts analysed and by inclusion of transcripts from all three classes. Available information about specific working alliances in the qualitative data were limited, hence missing other interesting themes. Although, over-sampling the interviews from participants could have improved the qualitative analyses, it was not possible to undertake due to limited resources for this study.

The study also had several strengths. It added to a body of evidence showing that professional helping relationships can have positive effects in severely disadvantaged groups [20, 21]. The analysis overcame the methodological limitations associated with previous studies (e.g., 19) including the absence of control groups, brief follow ups, and assumptions of sample homogeneity. Sample selection for the qualitative analysis was driven by the quantitative analysis. Further, this was one of the first studies to firmly establish the impact of the working alliance on the trajectories of people facing severe and multiple disadvantage in the context of a multisite randomised controlled trial of a major housing intervention. The effect of the working alliance on housing stability across the population and on its subgroups and the factors that may explain these differences have important implications for future responses to people facing life’s greatest challenges.

## Conclusions

The quality of professional helping relationships made a significant contribution to the housing stability of people experiencing homelessness and mental illness, with different effects detected on unique subgroups within the homeless population. Attention to structural and individual factors may ensure that more people benefit from the relationships developed with the workers charged with their support.

## Data Availability

The data that support the findings of this study are available from St. Michael’s Hospital, Unity Health Toronto but restrictions apply to the availability of these data, which were used under license for the current study, and so are not publicly available. Data are however available from the authors upon reasonable request and with permission of St. Michael’s Hospital, Unity Health Toronto.

## Abbreviations

ABIC: Sample-size adjusted BIC
ACE: Adverse Childhood Experiences
ACT: Assertive community treatment
AIC: Akaike information criterion
BIC: Bayesian Information Criterion
BLRT: Bootstrapped Likelihood Ratio test
CFI: Comparative Fit Index
CSI: Colorado Symptom Index
DF: Degrees of freedom
GMM: Growth mixture modelling
HS: Housing stability
Gain-SPS: Global Assessment of Individual Need Substance Problem Scale
ICM: Intensive case management
INT: Intercept
INTER: Intervention
LGCM: Latent growth curve model
LGMM: Latent growth mixture model
LCGA: Latent class growth analysis
LMR-LRT: Lo-Mendell-Rubin Likelihood Ratio test
MCAS: Multnomah Community Ability Scale
MINI: Mini-International Neuropsychiatric Interview
NNFI: Non-Normed Fit index
RTLFB: The Residential Time-Line Follow-Back Inventory
SRMSR: Standardized Root Mean Square Residual
RMSEA: Root Mean Square Error of Approximation
TAU: Treatment as usual
TVC: Time-varying covariate
TLI: Tucker-Lewis index
TIC: Time-invariant covariate
QUAD: Quadratic growth factor
WA: Working alliance
